# Expression Analysis of Cnidarian-Specific Neuropeptides in a Sea Anemone Unveils an Apical-Organ-Associated Nerve Net That Disintegrates at Metamorphosis

**DOI:** 10.3389/fendo.2020.00063

**Published:** 2020-02-19

**Authors:** Hannah Zang, Nagayasu Nakanishi

**Affiliations:** ^1^Lyon College, Batesville, AR, United States; ^2^Department of Biological Sciences, University of Arkansas, Fayetteville, AR, United States

**Keywords:** neuropeptide, evolution, cnidaria, sea anemone, neural development, metamorphosis

## Abstract

Neuropeptides are ancient neuronal signaling molecules that have diversified across Cnidaria (e.g., jellyfish, corals, and sea anemones) and its sister group Bilateria (e.g., vertebrates, insects, and worms). Over the course of neuropeptide evolution emerged lineage-specific neuropeptides, but their roles in the evolution and diversification of nervous system function remain enigmatic. As a step toward filling in this knowledge gap, we investigated the expression pattern of a cnidarian-specific neuropeptide—RPamide—during the development of the starlet sea anemone *Nematostella vectensis*, using *in situ* hybridization and immunohistochemistry. We show that RPamide precursor transcripts first occur during gastrulation in scattered epithelial cells of the aboral ectoderm. These RPamide-positive epithelial cells exhibit a spindle-shaped, sensory-cell-like morphology, and extend basal neuronal processes that form a nerve net in the aboral ectoderm of the free-swimming planula larva. At the aboral end, RPamide-positive sensory cells become integrated into the developing apical organ that forms a bundle of long cilia referred to as the apical tuft. Later during planula development, RPamide expression becomes evident in sensory cells in the oral ectoderm of the body column and pharynx, and in the developing endodermal nervous system. At metamorphosis into a polyp, the RPamide-positive sensory nerve net in the aboral ectoderm degenerates by apoptosis, and RPamide expression begins in ectodermal sensory cells of growing oral tentacles. In addition, we find that the expression pattern of RPamide in planulae differs from that of conserved neuropeptides that are shared across Cnidaria and Bilateria, indicative of distinct functions. Our results not only provide the anatomical framework necessary to analyze the function of the cnidarian-specific neuropeptides in future studies, but also reveal previously unrecognized features of the sea anemone nervous system—the apical organ neurons of the planula larva, and metamorphosis-associated reorganization of the ectodermal nervous system.

## Introduction

Neuropeptides are short polypeptide hormones that are generated from a larger precursor protein via proteolytic cleavage in neurons and neuroendocrine cells [reviewed in (1)]. The cleaved peptides, in some cases, undergo peptide α-amidation involving post-translational conversion of a C-terminal glycine into an amide group. Processed neuropeptides are stored in membrane-bound secretory vesicles that are distributed throughout the cell. Upon receiving stimuli, the secretory vesicles fuse with the cell membrane, releasing neuropeptides into the extracellular space and/or circulation. The neuropeptides then diffuse and bind to receptors—typically G-protein-coupled receptors—in the cell membrane of target cells, triggering intracellular signaling cascades. Neuropeptides, but not small molecule neurotransmitters such as glutamate, GABA and acetylcholine, are commonly found in the nervous systems of cnidarians (e.g., jellyfishes and sea anemones) and bilaterians (e.g., vertebrates and insects), and thus are thought to be the first neurotransmitter molecules in nervous system evolution (2, 3). In an attempt to better understand how the primordial nervous system may have functioned, efforts have been directed toward resolving deeply conserved functions of neuropeptides that are shared across Cnidaria and Bilateria. These studies suggest a likely ancestral role of deeply conserved Wamide neuropeptides in modulating life cycle transition (4–6). Comparably little is known, however, about how lineage-specific neuropeptides contribute to the evolution and diversification of nervous system function, particularly in Cnidaria. As a step toward understanding the role of lineage-specific neuropeptides in the evolution of neural function in Cnidaria, we investigated the expression pattern of a cnidarian-specific neuropeptide—RPamide—during development of the starlet sea anemone *Nematostella vectensis*.

Cnidaria is an early-evolving animal group that diverged from its sister group Bilateria over 600 million years ago in the Precambrian (7–11). Cnidaria represents a diverse clade in which Medusozoa forms a sister group to Anthozoa (12, 13); Medusozoa consists of Hydrozoa (e.g., Portuguese man o' war), Staurozoa (stalked jellyfish), Scyphozoa (e.g., moonjelly), and Cubozoa (e.g., sea wasp), and Anthozoa comprises Octocorallia (e.g., sea fans) and Hexacorallia (e.g., hard corals and sea anemones). Cnidarians are diploblastic, being composed of ectoderm and endoderm separated by an extracellular matrix called the mesoglea, and are characterized by having a phylum-defining stinging cell type, the cnidocyte. Cnidarian development typically entails multiple life cycle stages, whereby gastrulation generates a two-layered, free-swimming planula larva that metamorphoses into a sessile polyp with oral tentacles. The polyp sexually matures in Anthozoa, Staurozoa and some hydrozoan cnidarians (e.g., *Hydra*); in most medusozoans, the polyp undergoes another round of metamorphosis via transverse fission/strobilation (in Scyphozoa and Cubozoa) or lateral budding (in Hydrozoa) to generate free-swimming medusae that grow and reach sexual maturity. The nervous system develops in the ectoderm [and, in some cases, in the endoderm; e. g., the sea anemone *N. vectensis* (14, 15), the hydrozoan *Hydra littoralis* (16), and the scyphozoan *Cyanea capillata* (17)] at each of the life cycle stages [but see (18) for electron microscopic evidence for the lack of the nervous system in cubozoan planulae]. It is composed of epithelial sensory cells and basally localized ganglion cells, which extend basal neurites that form a basiepithelial network (19). This basic organization is modified to generate neural structures of varying morphological complexities, from the oral nerve ring of *Hydra* (20) to the lensed eyes of cubozoan medusae (21). Neuropeptides, but not small molecule neurotransmitters, are ubiquitously expressed in cnidarian nervous systems, and thus are thought to be the primary neurotransmitters and neurohormones of Cnidaria [reviewed in (2, 3)].

Among cnidarians, the starlet sea anemone *N. vectensis* is a useful model to examine neuropeptide function during development, because genomic and transcriptomic resources (8, 22, 23), molecular genetic tools [e.g., *in situ* hybridization and CRISPR-mediated mutagenesis (24, 25)], and data on neural anatomy and development (14, 15) are available. In *N. vectensis*, gastrulation occurs by invagination (26, 27), and the site of gastrulation—the blastopore—becomes the mouth of the animal (28). Both sensory cells and ganglion cells begin to develop in the outer ectoderm during gastrulation (14, 29). The gastrula embryo then develops into a free-swimming planula larva that forms a bundle of long cilia at the aboral pole, which is referred to as the apical tuft (14, 30). The ectodermal structure that houses the apical tuft is termed the apical organ, and is often assumed to be a sensory structure that is used to perceive sensory signals for settlement and metamorphosis [e.g., (30)]. Early-born neurons in the ectoderm send basal neurites toward the base of the apical organ, forming a basiepithelial nerve net (14). During planula development, sensory cells develop in the pharyngeal ectoderm, as well as in the endoderm (14). At metamorphosis, the planula larva transforms into a sessile polyp by developing circumoral tentacles with mechanosensory hair cells and losing the apical tuft (14). We note that *N. vectensis* neurons have been reported to show diverse transcriptome profiles (31), and thus transcriptionally distinct neural cell types may exist among morphologically similar neurons.

Isolated originally from the green aggregating anemone *Anthopleura elegantissima* (32, 33) and recently from the starlet sea anemone *N. vectensis* (34), RPamide represents one of the cnidarian-specific peptides. RPamide precursor-encoding genes have been recovered from the genomes of anthozoans [*A. elegantissima* (35) and *N. vectensis* (36); but absent in Octocorallia (37)] and medusozoans [hydrozoans *Clytia hemisphaerica* and *Cladonema pacificum* (38, 39); scyphozoans *Nemopilema nomurai* and *Rhopilema esculentum* (37); but absent in staurozoans and cubozoans (37)], but not from those of other metazoan groups. Immunohistochemical evidence suggests that RPamide is a neuropeptide; immunostaining with an antiserum against RPamide has shown that RPamide is expressed in ectodermal sensory cells of the body wall in the sea anemone *Calliactis parasitica* (32) and in ectodermal photosensory cells in gonads of the hydrozoan *C. hemisphaerica* (38). Treatment of isolated sea anemone tentacles with synthetic RPamide changed the rates of spontaneous contractions, consistent with a role in neurotransmission (32, 33). In addition, recent experimental evidence suggests that RPamide regulates oocyte maturation and spawning in hydrozoans (39). However, whether RPamide has any role during cnidarian development is not known.

In this paper, we have investigated the developmental expression pattern of RPamide in *N. vectensis* in order to build a neuroanatomical framework for understanding RPamide function during development. By using *in situ* hybridization and immunohistochemistry, we show that RPamide is dynamically expressed during *N. vectensis* development. RPamide expression begins at gastrulation in scattered epithelial cells in the aboral ectoderm. These RPamide-positive epithelial cells show a spindle-shaped, sensory cell-like morphology and extend basal neurites that form an aboral nerve net of the planula larva. A subset of the RPamide-positive sensory cells located at the aboral end become integrated into the developing apical organ. Later during planula development, RPamide-positive sensory cells become evident in oral ectoderm of the body column and the pharynx, as well as in the endoderm. At metamorphosis, RPamide-positive aboral sensory nerve net disintegrates by apoptosis, and RPamide becomes expressed in ectodermal sensory cells of growing oral tentacles. In addition, we find that expression of RPamide and that of the conserved neuropeptide GLWamide occurs in distinct subsets of neurons in planulae, suggesting that RPamide and GLWamide may perform non-overlapping functions in *N. vectensis* planulae.

## Materials and Methods

### Animal Culture

*N. vectensis* were cultured as previously described (40, 41).

### RNA Extraction, cDNA Synthesis, and Gene Cloning

Total RNA was extracted from a mixture of planulae and primary polyps using TRIzol (Thermo Fisher Scientific). cDNAs were synthesized using the BD SMARTer RACE cDNA Amplification Kit (Cat. No. 634858; BD Biosciences, San Jose, CA, USA). RPamide gene sequences [Nv37852 and Nv244953; (36)] were retrieved from the Joint Genome Institute genome database (*N. vectensis* v1.0; http://genome.jgi-psf.org/Nemve1/Nemve1.home.html). 5′ and 3′ RACE were conducted, following manufacturer's recommendations (BD SMARTer RACE cDNA Amplification Kit, BD Biosciences, San Jose, CA, USA), in order to confirm *in silico* predicted gene sequences. Gene specific primer sequences used for RACE PCR are: 3′ RACE Forward 5′-GCTCGGTACAGAGCCGAAACCTGAGACAC-3′; 5′ RACE primary PCR Forward 5′-CATGGGCAACGGTCAGCGGCAGATCGATG-3′. RACE PCR fragments were cloned into the pGEM-T plasmid vector using the pGEM-T Vector Systems (Cat. No. A3600; Promega), and were sequenced at Macrogen Corp., Maryland. To generate templates for RNA probes for *in situ* hybridization experiments, RTPCR was performed to amplify a 406 bp NvRPa (Nv244953) gene fragment. Gene specific primer sequences used for RTPCR are: Forward 5′-CGAAGGACCTTGAAAGTGGACTGTTCTCGG-3′; Reverse 5′-CATGGGCAACGGTCAGCGGCAGATCGATG-3′. The PCR product was cloned into a pCR4-TOPO TA vector using the TOPO TA Cloning kit (Cat. No. K457501; ThermoFisher Scientific), and sequenced at Genewiz, New Jersey.

### Generation of an Antibody Against RPamide

**Figure 1:**
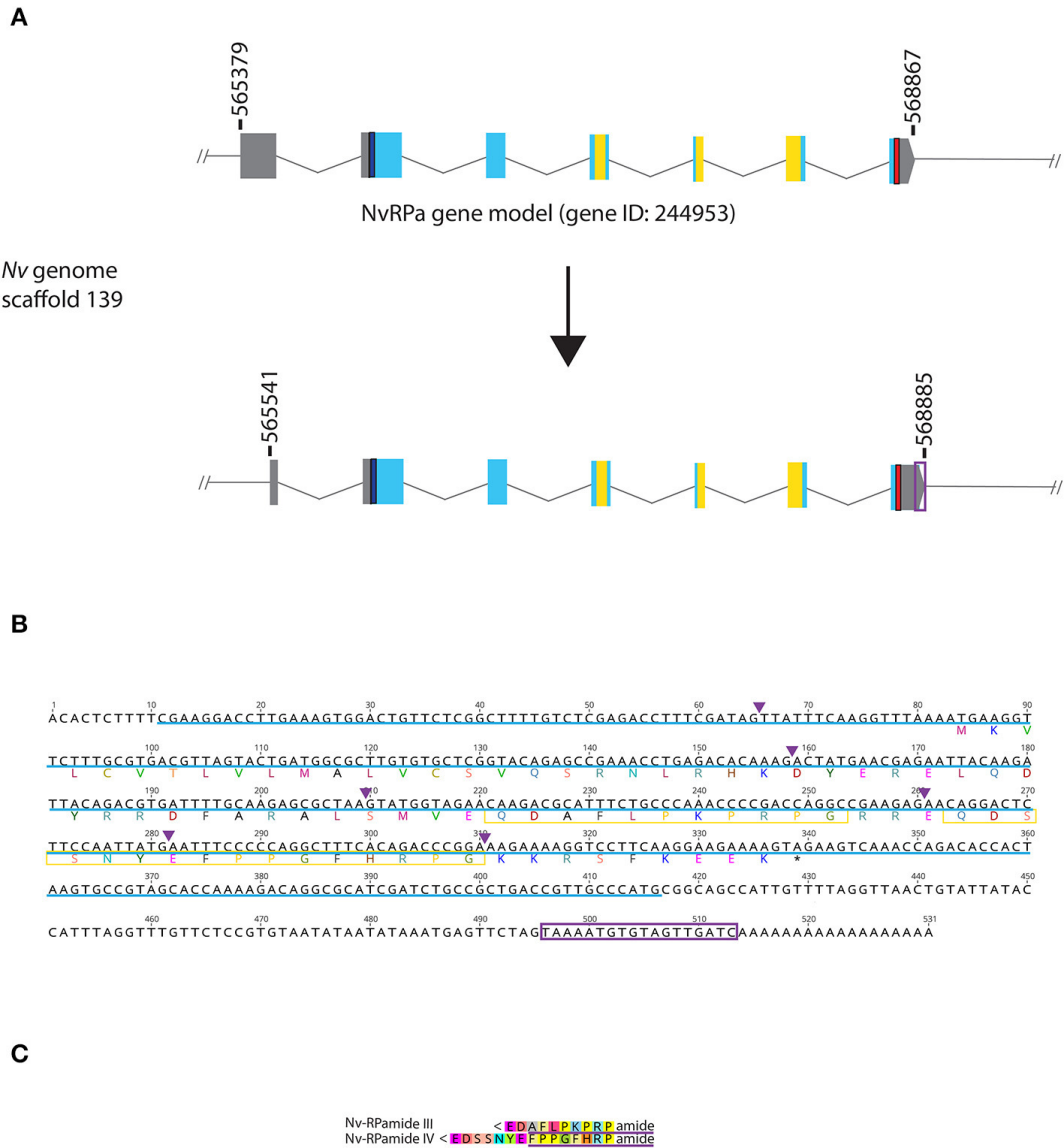
Genomic organization, cDNA sequence, and predicted mature peptides of RPamide gene. **(A)** Schematic view of the NvRPa genomic locus Nv244953 located in scaffolds 139 of the *N. vectensis* genome (v.1.0; http://genome.jgi.doe.gov/Nemve1/Nemve1.home.html). Gene structure based on gene model prediction is shown at the top, and the revised structure based on the comparison of the *N. vectensis* genome and cDNA sequences is shown at the bottom. The number above the schematic indicate genomic coordinates within the scaffold. Gray boxes are untranslated regions; aqua-colored boxes are translated regions. Blue bars show predicted translation start sites; red bars show predicted translation termination sites; yellow bars show predicted RPamide-encoding regions described below. Note that the revised model has a shorter exon 1 and a longer exon 7 (boxed in purple) relative to the predicted gene model. **(B)** cDNA and deduced amino acid sequence of NvRPa. Short peptides predicted to be released by proteolytic cleavages in a previous study by Anctil (36) are boxed in yellow. Single or multiple acidic and basic residues are assumed to become cleaved as suggested in another sea anemone *Anthopleura elegantissima* (32, 33, 35). Purple arrowheads indicate intron positions. The sequence used as a template to generate riboprobes for *in situ* hybridization is highlighted in light blue. **(C)** Amino acid sequence alignment for RPamide, “Nv-RPamide III,” and “Nv-RPamide IV”; nomenclature of these peptides was adopted from a previous genomic study (36). It is assumed that upon release of the peptide, an N-terminal glutamine is spontaneously converted into a pyroglutamic acid residue (<Glu), and a C-terminal glycine becomes amidated by an α-amidating enzyme [cf. (35)]. A single precursor protein is predicted to generate one copy of each peptide. We note the possibility that, given the presence of additional acidic residues within regions predicted to encode Nv-RPamide III and IV—namely, aspartic acid in Nv-RPamide III, and glutamic acid in Nv-RPamide IV—the processed peptides could be shorter; Nv-RPamide III may have the sequence AFLPKPRFamide and Nv-RPamide IV FPPGFHRPamide (purple underlines), the latter of which has been recently confirmed to occur in adult *N. vectensis* using mass spectrometry (34).

An antibody against a synthetic peptide CEDSSNYEFPPGFHRPamide corresponding in amino acid sequence to Nv-RPamide IV [*sensu* (36); Figure 1] was generated in rabbit (YenZym Antibodies, LLC); a recent mass spectrometry study has confirmed that the C-terminal FPPGFHRPamide is secreted by adult *N. vectensis* (34). The antibody was generated against the predicted Nv-RPamide IV sequence because mass spectrometry data were not available when the antibody was produced. Following immunization, the resultant antiserum was affinity purified with the CEDSSNYEFPPGFHRPamide peptide. The affinity purified antibody was then affinity-absorbed with KWSCSLRPamide and KWSCCLRPamide, which correspond to predicted RPamide peptides encoded by Nv37852—Nv-RPamide I and Nv-RPamide II, respectively [*sensu* (36)]—in order to generate a Nv-RPamide IV-specific antibody. Immunostaining with the antibody preadsorbed with the synthetic Nv-RPamide IV (CEDSSNYEFPPGFHRPamide) for 2 h at 37°C showed little immunoreactivity at the mid planula and primary polyp stages (Supplementary Figure 4), suggesting that the antibody reacts with Nv-RPamide IV. However, this antibody likely cross-reacts with other RPamide-like peptides, as we have observed a population of neurons that are immunoreactive with the antibody but do not express NvRPa transcripts in the tentacular ectoderm at the tentacle-bud and primary polyp stages (e.g., **Figure 4C**).

**Figure 2:**
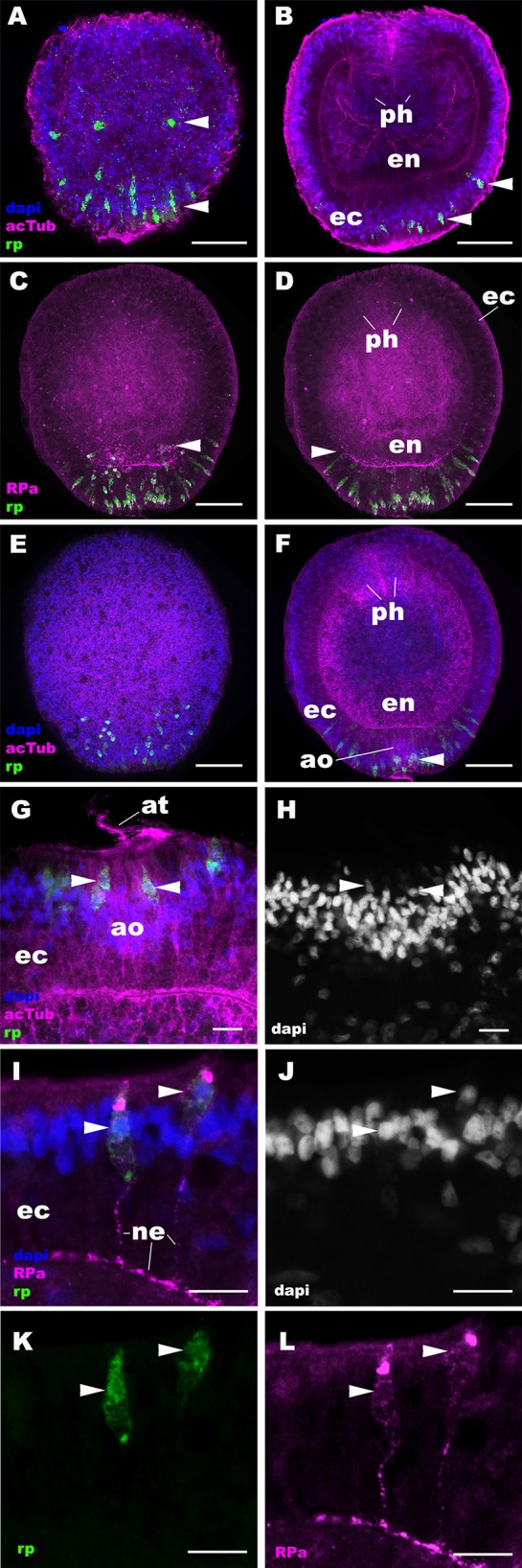
RPamide-positive sensory epithelial cells form a nerve net in the aboral ectoderm during gastrulation and early planula development in a sea anemone. Z-projections of confocal sections of *Nematostella vectensis* at gastrula **(A,B)** and early planula **(C–L)** stages, labeled with an antisense riboprobe against NvRPa transcript (“rp”) and antibodies against RPamide (“RPa”) and acetylated ∂-tubulin (“acTub”). Nuclei are labeled with DAPI. **(A–F)** are side views of animals with the blastopore/oral opening facing up. **(A,C,E)** show superficial planes of section at the level of the ectoderm, and **(B,D,F)** show longitudinal sections through the center of the animal. **(G,H)** depict an apical organ (ao) and aboral-lateral ectoderm, respectively; apical surface is to the top. During gastrulation, scattered epithelial cells in the aboral ectoderm begin to express NvRPa transcripts (arrowheads in **A,B**), which show spindle-shaped morphologies characteristic of cnidarian sensory cells (19). At the early planula stage, NvRPa-expressing sensory cells extend basal RPamide-positive neurites forming an aboral basiepithelial nerve net (arrowheads in **C,D**), and a subset of NvRPa-expressing sensory cells become integrated into the developing apical organ (ao; arrowhead in **F**). Note that the cell bodies of NvRPa-expressing sensory cells are located in the superficial stratum of the apical organ (arrowheads in **G,H**) and of the lateral ectoderm (arrowheads in **I–L**). ph, pharynx; ec, ectoderm; en, endoderm; at, apical tuft; ne, neurite. Scale bar: 50 μm **(A–F)**; 10 μm **(G–L)**.

### CRISPR-Cas9-Mediated Mutagenesis

20 nt-long sgRNA target sites were manually identified in the genomic locus for NvRPa. To minimize off-target effects, target sites that had 17 bp-or-higher sequence identity elsewhere in the genome (*N. vectensis v1.0*; http://genome.jgi.doe.gov/Nemve1/Nemve1.home.html) were excluded. Selected target sequences were as follows.

5′-ACTGATGGCGCTTGTGTGCT-3′ (Cr1)

5′-CTATTCGGCTTTATTAGGTA-3′ (Cr2)

5′-GGTATAACTATCCCGTTCCT-3′ (Cr3)

The sgRNA species were synthesized *in vitro* (Synthego), and mixed at equal concentrations. The sgRNA mix and Cas9 endonuclease (PNA Bio, PC15111, Thousand Oaks, CA, USA) were co-injected into fertilized eggs at concentrations of 500 ng/μl and 1,000 ng/μl, respectively.

### Genotyping of Embryos

Genomic DNA from single embryos was extracted by using a published protocol (24), and the targeted genomic locus was amplified by nested PCR. Primer sequences used for nested genomic PCR are: “1” Forward 5′-CGAAGGACCTTGAAAGTGGACTGTTCTCGG-3′, “1” Reverse 5′-TGTCTGGGACTAGTTTACCTACAGCG-3′, “2” Forward 5′-GTGGTATGAGGCACAAACGTAGATGG-3′, “2” Reverse 5′-CATGGGCAACGGTCAGCGGCAGATCGATG-3′.

### Immunofluorescence, TUNEL and *in situ* Hybridization

Animal fixation, immunohistochemistry, and *in situ* hybridization were performed as previously described (14, 25). For immunohistochemistry, we used primary antibodies against RPamide (rabbit, 1:200), GLWamide [rabbit, 1:200; (5)], acetylated ∂-tubulin (mouse, 1:500, Sigma T6793) and tyrosinated ∂-tubulin (mouse, 1:500, Sigma T9028), and secondary antibodies conjugated to AlexaFluor 568 [1:200, Molecular Probes A-11031 (anti-mouse) or A-11036 (anti-rabbit)] or AlexaFluor 647 [1:200, Molecular Probes A-21236 (anti-mouse) or A-21245 (anti-rabbit)]. Nuclei were labeled using fluorescent dyes DAPI (1:1,000, Molecular Probes D1306), and filamentous actin was labeled using AlexaFluor 488-conjugated phalloidin (1:25, Molecular Probes A12379). TUNEL assay was carried out after immunostaining, by using *in situ* Cell Death Detection Kit (TMR red cat no. 1684795, Roche, Indianapolis, IN, USA) or Click-iT Plus TUNEL Assay for *in situ* Apoptosis Detection (Alexa Fluor 488, cat no. C10617, Molecular Probes) according to manufacturer's recommendation; both TUNEL assay kits showed similar results, and thus only data generated by using the Roche kit are reported. For *in situ* hybridization, antisense and sense digoxigenin-labeled riboprobes were synthesized by using the MEGAscript transcription kits according to manufacturer's recommendation (Ambion; T7, AM1333; T3, AM1338), and were used at the final concentration of 1 ng/μl. Fluorescent images were recorded using a Leica SP5 Confocal Microscope or a Zeiss LSM900. Images were viewed using ImageJ.

## Results

It has been previously reported that the genome of the starlet sea anemone *N. vectensis* contains two RPamide-precursor genes [Nv37852 and Nv244953; (36)]. However, Reverse Transcriptase PCR by using planula and polyp cDNAs showed detectable expression for Nv244953, but not for Nv37852 (Supplementary Figure 1). Thus, we have focused our present analyses on Nv244953, which we will herein refer to as NvRPa (N*ematostella* v*ectensis* RPamide). Consistent with *in silico* gene prediction (https://mycocosm.jgi.doe.gov/cgi-bin/dispGeneModel?db$=$Nemve1&id$=$244953), comparison of the *N. vectensis* genome and NvRPa cDNA sequence indicates that NvRPa is a 7-exon gene. We note that the cDNA sequence has a shorter exon 1 and a longer exon 7 relative to the *in silico* predicted gene model (Figures 1A,B); however, the shorter exon 1 could have resulted from failure to synthesize full-length cDNA.

Based on the location of diagnostic endoproteolytic cleavage sites (i.e., acidic residues, aspartic/glutamic acid; basic residues, lysine/argenine) and C-terminal amidation sites (i.e., glycine residue) [reviewed in (2)], NvRPa is predicted to encode single copies of two RPamide peptides that differ in N-terminal sequence [Figure 1C; Nv-RPamide III and IV, *sensu* (36)].

### RPamide Is Expressed in the Aboral Sensory Nerve Net of the Planula Larva

Next we examined the spatiotemporal expression patterns of NvRPa during *N. vectensis* development. We used *in situ* hybridization and immunostaining to localize NvRPa transcripts and RPamide peptides, respectively. *In situ* hybridization using a sense probe did not result in cell-type-specific staining (Supplementary Figure 2). An antibody against RPamide peptides was made against Nv-RPamide IV predicted to be generated by NvRPa (see section Materials and Methods), and was validated by CRISPR-Cas9-mediated mutagenesis; a significantly reduced average number of anti-RPamide-immunoreactive neurons was observed in NvRPa F0 mosaic mutants (2; *n* = 5) relative to the wildtype control (20; *n* = 5) (two tailed *t*-test, *p* < 0.0001; Supplementary Figure 3). In addition, immunostaining with the antibody that was preadsorbed with Nv-RPamide IV peptides did not result in cell-type-specific staining (Supplementary Figure 4), indicating that the antibody indeed reacts with Nv-RPamide IV. *In situ* hybridization shows that NvRPa transcripts first occur at gastrulation in scattered epithelial cells of the aboral ectoderm, which exhibit a spindle-shaped morphology characteristic of cnidarian sensory cells (arrowheads in Figures 2A,B). During early planula development, these NvRPa-expressing epithelial cells extend RPamide-positive basal neuronal processes that form an interconnected basiepithelial network in the aboral ectoderm (Figures 2C,D). A subset of NvRPa-expressing sensory cells become integrated into the apical organ developing at the aboral end (Figures 2E–H). The cell bodies of RPamide-positive neurons are located in the superficial stratum of the ectoderm (arrowheads in Figures 2G–L). Consequently, within the apical organ, RPamide-positive sensory cells can be distinguished from apical-tuft cells based on the position of nuclei; the former have superficial nuclei, while the latter have basal nuclei [Figures 2G,H; (14)]. Thus, RPamide appears active in the aborally concentrated sensory nerve net of the planula larva.

**Figure 3:**
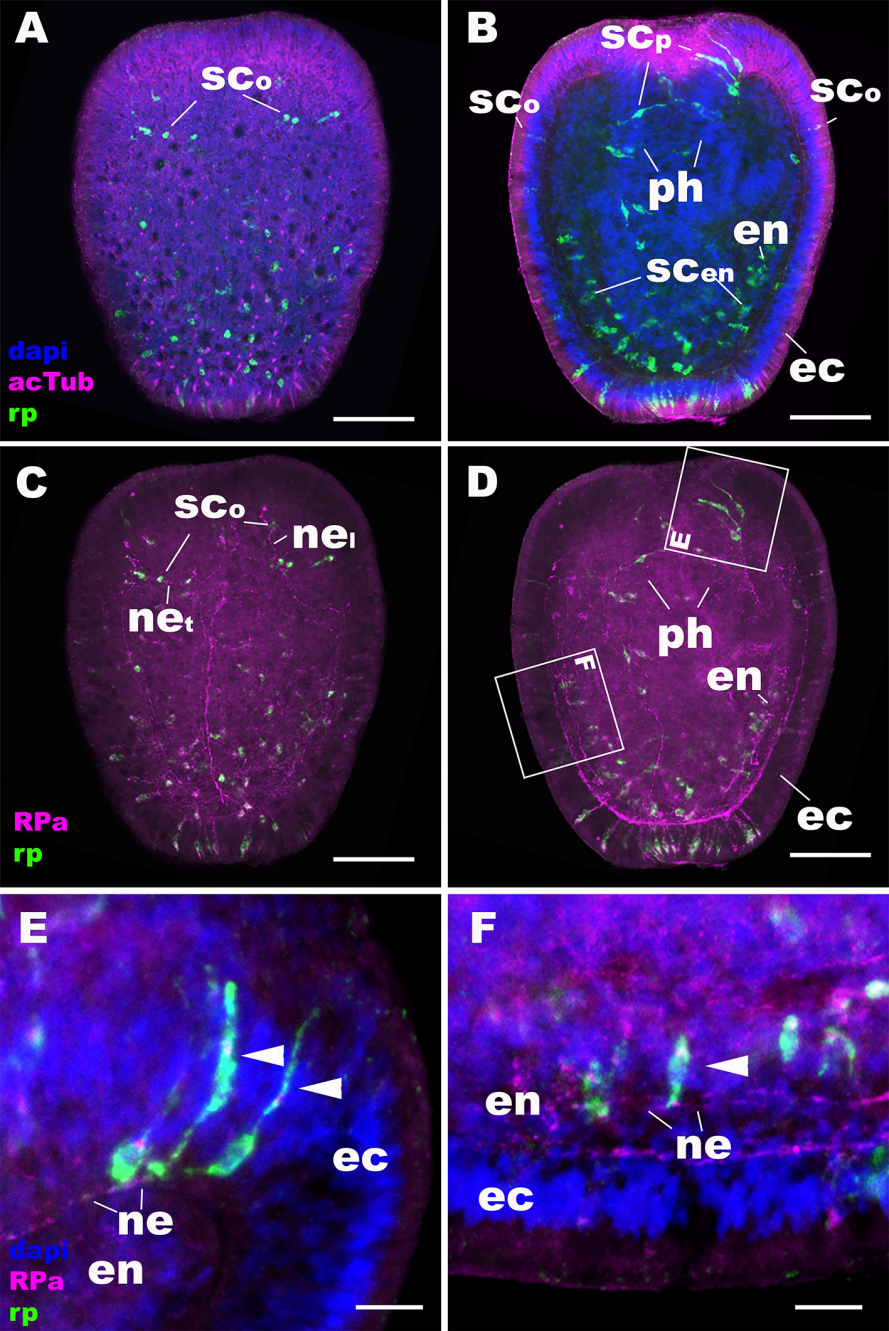
During sea anemone planula development, RPamide becomes expressed in the nervous system of the oral body column ectoderm, pharyngeal ectoderm, and endoderm. Z-projections of confocal sections of *Nematostella vectensis* at mid-planula stages, labeled with an antisense riboprobe against NvRPa transcript (“rp”) and antibodies against RPamide (“RPa”) and acetylated ∂-tubulin (“acTub”). Nuclei are labeled with DAPI. **(A–D)** are side views of animals with the oral opening facing up. **(A,C)** show superficial planes of section at the level of the ectoderm; **(B,D)** show longitudinal sections through the center. **(E,F)** are magnified views of boxed regions in **(D)** showing NvRPa-expressing cells with their apical side facing up. During planula development, NvRPa becomes expressed in epithelial cells with the sensory-cell-like morphology and RPa-positive basal neurites. These NvRPa-expressing sensory cells reside in the body column oral ectoderm (sc_o_ in **A–C**), pharyngeal ectoderm (sc_p_ in B; arrowheads in **E**) and endoderm (sc_en_ in **B**; an arrowhead in **F**). Note in C that NvRPa-expressing sensory cells in the body column oral ectoderm can have aborally oriented longitudinal neurites (ne_l_), or transverse neurites (ne_t_). NvRPa-expressing sensory cells in the pharynx have aborally oriented longitudinal neurites (ne in **E**), and those in the endoderm send longitudinal neurites in both oral and aboral directions (ne in **F**). ph, pharynx; ec, ectoderm; en, endoderm. Scale bar: 50 μm **(A–D)**; 10 μm **(E,F)**.

At later stages of planula development, NvRPa becomes expressed in sensory cells of the oral body column ectoderm, pharyngeal ectoderm, as well as in the developing endodermal nervous system (Figures 3A,B). Some NvRPa-expressing sensory cells of the oral ectoderm extend basal neurites in an aboral direction (ne_l_ in Figure 3C). while others extend in a transverse orientation (ne_t_ in Figure 3C). NvRPa-expressing neurons in the pharyngeal ectoderm extend neurites preferentially in an aboral direction (Figures 3D,E). In the endoderm, NvRPa expression primarily occurs in neurons with longitudinally oriented neurites (Figures 3D,F), which become part of longitudinal neuronal bundles of the polyp endodermal nervous system (see below).

### RPamide-Positive Ectodermal Nervous System Is Reorganized at Metamorphosis, Involving Removal of the Aboral Sensory Nerve Net by Apoptosis, and *de novo* Development of RPamide-Positive Tentacular Sensory Cells

**Figure 4:**
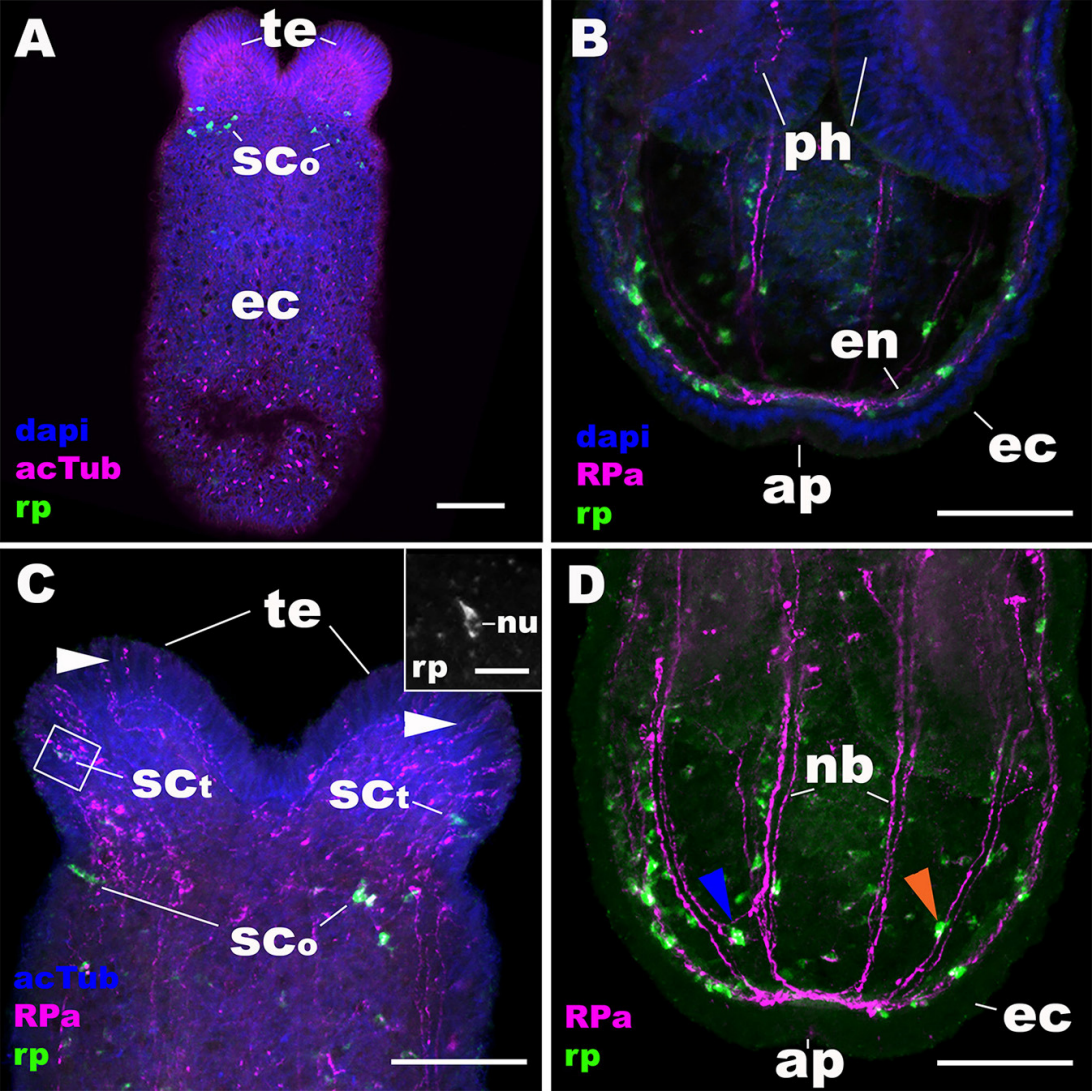
During metamorphosis of a planula larva into a sea anemone polyp, the RPamide-positive sensory nerve net of the aboral ectoderm disappears, and RPamide becomes expressed in ectodermal sensory cells of developing oral tentacles. Z-projections of confocal sections of *Nematostella vectensis* at the tentacle-bud/primary polyp stage, labeled with an antisense riboprobe against NvRPa transcript (“rp”) and antibodies against RPamide (“RPa”) and acetylated ∂-tubulin (“acTub”). Nuclei are labeled with DAPI. All panels show side views of the animal with the oral opening facing up. **(A,C)** show superficial planes of section at the level of surface ectoderm (ec); **(B,D)** show longitudinal sections through the center. **(B,D)** depict an aboral half of the animal, while **(C)** shows an oral half of the animal. The inset in **(C)** is a magnified view of the boxed region showing a NvRPa-expressing cell with their apical side facing up; note that NvRPa transcripts primarily localize to the cytoplasm so that the nucleus (nu) appears NvRPa-negative. At metamorphosis, RPamide expression becomes undetectable in the aboral ectoderm at the transcript and peptide levels (compare **A,B** with Figures 2A–D). Concomitantly, NvRPa transcripts become expressed in ectodermal sensory cells of the developing oral tentacles that send RPamide-positive longitudinal neurites in an aboral direction (sc_t_ in **C**); note in the inset in **(C)** the sensory cell-like spindle-shaped morphology of an NvRPa-expressing tentaclular cell. White arrowheads in **(C)** show anti-RPamide-immunoreactive sensory cells in the tentacular ectoderm that do not express detectable levels of NvRPa transcripts, which suggests that the immunoreactive materials in these cells are not produced by NvRPa. Note in **(D)** that RPamide expression is evident in endodermal neurons that are integrated within longitudinal neuronal bundles (nb) (orange arrowhead), as well as in those that reside between neighboring neuronal bundles (blue arrowhead); neurites from the latter neurons connect with those from neurons in the neuronal bundles. te, tentacle; sc_o_, sensory cell in the oral body column ectoderm; ph, pharynx; en, endoderm; ap, aboral pole. Scale bar: 50 μm **(A–D)**; 10 μm (inset in **C**).

At metamorphosis, the planula larva transforms into a polyp by developing tentacles around the mouth. During this process, the NvRPa-expressing aboral sensory nerve net disappears (Figures 4A,B) along with the apical organ (14). Concomitantly, a subset of sensory cells in the tentacular ectoderm begin to express NvRPa (SC_t_ in Figure 4C). In addition, NvRPa expression is detected in some of the endodermal neurons with transverse neurites that connect with longitudinal neuronal bundles (blue arrowhead in Figure 4D). We note that at this developmental stage (i.e., tentacle bud and primary polyp stages) we observed a population of neurons that are immunoreactive with the antibody but do not express NvRPa transcripts in the tentacular ectoderm (Figure 4C); over 92% of RPa-immunoreactive neurons in oral tentacles (*n* = 77) did not show detectable levels of NvRPa expression. This suggests that RPamide-like peptides that are not generated by NvRPa are expressed in some of the tentacular ectodermal neurons. Alternatively, NvRPa could be expressed in these neurons at levels not detectable by *in situ* hybridization. The lack of co-localization of NvRPa transcripts and RPa-immunoreactivity is rarely found prior to metamorphosis.

**Figure 5:**
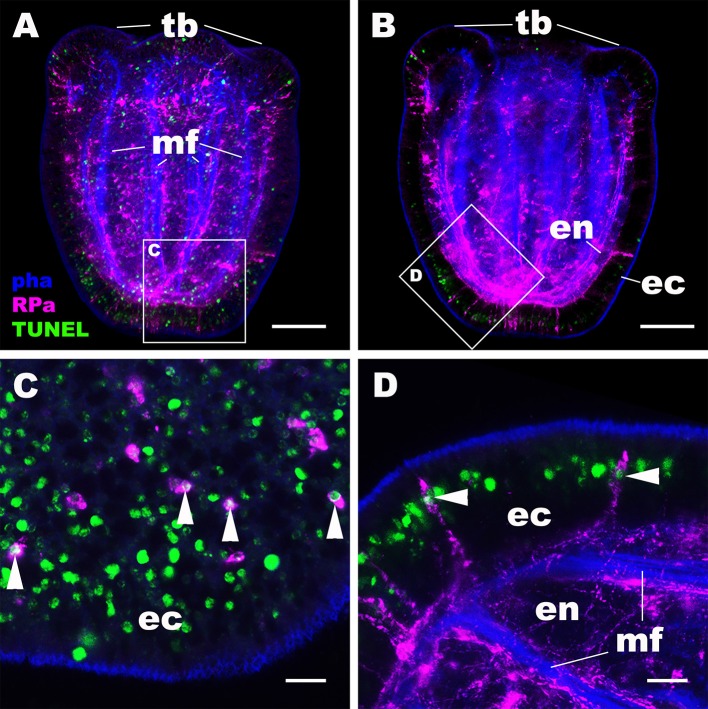
RPamide-positive sensory cells of the aboral ectoderm undergo apoptosis at metamorphosis. Z-projections of confocal sections of *Nematostella vectensis* at the tentacle-bud stage, labeled with an antibody against RPamide (“RPa”). Filamentous actin is labeled with a fluorescent dye phalloidin (“pha”), and DNA fragmentation is detected by TUNEL assay (“TUNEL”). **(A,B)** are side views of the animal with the oral opening facing up; **(A)** represents a superficial plane of section at the level of surface ectoderm and parts of the endoderm, while B depicts a longitudinal section through the center. **(C,D)** are sections from boxed areas in **(A,B)**, respectively; note in D that the orientation has been altered relative to B so that the apical surface of the ectoderm faces up. Arrowheads in **(C,D)** point to TUNEL-positive DNA fragmentation in RPamide-positive sensory cells of the aboral ectoderm, evidencing that these cells are undergoing programmed cell death. tb, tentacle bud; mf, endodermal muscle fibers; ec, ectoderm; en, endoderm. Scale bar: 50 μm **(A,B)**; 10 μm **(C,D)**.

We then examined the mechanism by which the NvRPa-expressing aboral sensory nerve net was removed at metamorphosis in *N. vectensis*. Specifically, we have considered the possibility that NvRPa-expressing aboral sensory cells undergoes programmed cell death at metamorphosis. To test this hypothesis, we labeled apoptotic DNA fragmentation at metamorphosis by using the terminal deoxynucleotidyl transferase dUTP nick end-labeling (TUNEL) assay. We have found that, indeed, NvRPa-expressing aboral sensory cells contain TUNEL-positive nuclei and/or nuclear fragments at the tentacle-bud stage (69.6%, *n* = 46; Figure 5). This finding supports the hypothesis that the NvRPa-expressing sensory nerve net in the aboral ectoderm of the planula larva disintegrates by apoptosis during metamorphosis in *N. vectensis*. We note that apoptosis in the aboral ectoderm at metamorphosis is not restricted to RPamide-positive neurons, but include other peptidergic neurons such as GLWamide- and RFamide-immunoreactive neurons (Supplementary Figure 5).

### The Expression Pattern of RPamide Is Distinct From That of GLWamide

**Figure 6:**
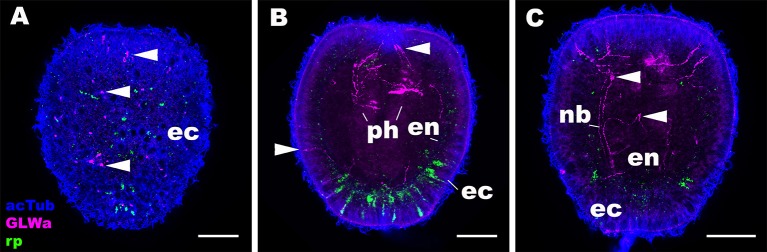
RPamide and GLWamide are expressed in distinct, non-overlapping subsets of sensory cells in the ectoderm of a sea anemone planula larva. Z-projections of confocal sections of *Nematostella vectensis* at the mid-planula stage, labeled with an antisense riboprobe against NvRPa transcript (“rp”) and antibodies against GLWamide (“GLWa”) and acetylated ∂-tubulin (“acTub”). All panels show side views of animals with the oral opening facing up. **(A)** shows a superficial plane of section at the level of surface ectoderm (ec). **(B,C)** Show longitudinal sections at the level of pharynx (ph) and longitudinal neuronal bundles (nb), respectively. Arrowheads point to GLWamide-positive neurons that are NvRPa-negative, which include sensory cells in the body column ectoderm **(A,B)**, in pharyngeal ectoderm **(B)**, and in endoderm (en; **C**). Note also that none of the NvRPa-expressing cells are GLWamide-positive. Scale bar: 50 μm.

Next we asked whether the expression pattern of NvRPa was distinct from that of GLWamide—a neuropeptide thought to be conserved across Cnidaria and Bilateria, which regulates the timing of life cycle transition in *N. vectensis* (5). To address this, we combined *in situ* hybridization and immunostaining to examine the spatial localization of NvRPa transcripts and GLWamide peptides during planula development in *N. vectensis*. We found no evidence of colocalization of NvRPa and GLWamide expression in ectodermal and endodermal nervous systems in *N. vectensis* planulae (Figure 6). Thus, RPamide and GLWamide are expressed in non-overlapping subsets of neurons in the planula nervous systems in *N. vectensis*, suggesting that RPamide and GLWamide may perform non-overlapping neural functions in planulae. These data are also consistent with multiple peptidergic neuron types having complementary roles in specific biological processes such as metamorphosis.

## Discussion

In this paper, we reported the expression pattern of a cnidarian-specific neuropeptide, RPamide, during development of the starlet sea anemone *N. vectensis*. We have found that early RPamide-positive neurons form a sensory nerve net in the aboral ectoderm of the planula larva, and that a subset of RPamide-positive sensory cells located at the aboral end become part of the apical organ. During planula development, RPamide becomes expressed in sensory cells of oral ectoderm in the body column and pharynx, as well as in endodermal neurons. At metamorphosis, the RPamide-positive sensory nerve net in aboral ectoderm disappears by apoptosis, and RPamide becomes expressed in a subset of ectodermal sensory cells in growing oral tentacles. RPamide expression and GLWamide expression do not co-occur in the same neurons in planulae, indicative of functional segregation between the two neuropeptides.

### The Sea Anemone Apical Organ Consists of Multiple Cell Types, Including Apical Tuft Cells and Peptidergic Sensory Cells

Our study sheds light on the diversity of cell types that constitute the apical organ in a sea anemone planula. Chia and Koss (30) used electron microscopy to describe the structure of an apical organ in the sea anemone *A. elegantissima*. They reported that the apical organ consists of columnar epithelial cells, each of which has a long cilium and a collar of microvilli on the apical cell surface and a basally localized nucleus. Long cilia from the columnar epithelial cells form a ciliary bundle known as the apical tuft, and their ciliary rootlet characteristically reach the basal processes of the cells. In addition, Nakanishi et al. (14) identified two types of gland cells in the apical organ of *N. vectensis*: one with electron-lucent granules and the other with electron-dense granules. In this study, we have discovered that a subset of early-born RPamide-positive sensory cells in the aboral ectoderm become part of the apical organ in *N. vectensis*. RPamide-positive sensory cells of the apical organ are morphologically distinct from other cell types that are housed in this structure. Specifically, in contrast to the apical-tuft epithelial cells that are column-shaped and have basal nuclei, these RPamide-positive sensory cells are spindle-shaped and contain nuclei located close to the epithelial surface. To our knowledge, this is the first evidence that the sea anemone apical organ contains a peptidergic sensory cell type. Thus, the apical organ is composed of at least three cell types: apical-tuft cells, gland cells, and peptidergic sensory cells.

Motivated by the question of whether the aboral domain of cnidarians is homologous to the anterior domain of bilaterians, several studies investigated the molecular mechanism of apical organ development in *N. vectensis* [e.g., (42–44)]. It has been proposed that apical organ development in *N. vectensis* occurs in two phases, involving a set of regulatory factors—*Six3, FoxQ2* and *FGFs*—whose bilaterian homologs play a conserved role in controlling anterior development (43). First, during gastrulation, *six3/6* specifies the identity of the broad aboral domain in the ectoderm by positively regulating the expression of *fgfa1* and *foxQ2a*. Subsequently, during planula development, FGFa1 signaling drives the differentiation of apical organ cells by downregulating *six3/6* and *foxQ2a* expression at the aboral pole. Consistent with this model, experimental perturbation of *six3/6* and FGFa1 expression results in planula larvae without apical tufts (42, 43). However, it has not been clear whether all apical organ cell types—besides the apical-tuft cells—develop in this way. In the present study, we have found that development of RPamide-positive sensory cells at the aboral end occurs during gastrulation, and precedes the mid-planula stage at which downregulation of *six3/6* is reported to occur (43). This suggests that neurogenesis that generates apical organ neurons takes place prior to differentiation of apical tuft cells, and therefore does not depend on downregulation of *six3/6* at the aboral pole. Our data therefore imply that neurogenesis and ciliogenesis in apical organ development are mechanistically decoupled. Consistent with this hypothesis, positive regulators of neurogenesis, such as *soxB(2), ath*, and *ashA*, are expressed in the aboral domain during gastrulation, prior to apical tuft formation (29, 45–47). How the mechanisms of neurogenesis and ciliogenesis in the context of sea anemone apical organ development differ from each other, and how they relate to anterior development of bilaterians, are to be addressed in future studies.

### Reorganization of the Ectodermal Nervous System at Metamorphosis Represents an Ancestral Condition in Cnidaria

Nervous system development in the sea anemone *N. vectensis* has been viewed as a gradual process that entails progressive addition of new features; neurons begin development in ectoderm at gastrulation, then in endoderm during planula development, and finally in the polyp tentacular ectoderm at metamorphosis (14, 15). Only apical tuft cells have been reported to disappear at metamorphosis in *N. vectensis* (14). In contrast, reorganization of peptidergic ectodermal nervous systems at the planula-polyp transition is commonly observed in non-sea anemone cnidarians such as the coral *Acropora* (48), moonjelly *Aurelia* (49), and hydrozoans [*Pennaria disticha* (formerly *Pennaria tiarella*) (50); *Hydractinia echinate* (51, 52); *Clava multicornis* (53)]. This process typically involves disappearance of the planula peptidergic nervous system—housed exclusively in the ectoderm in these cnidarians—likely via apoptosis (49, 53–56), followed by the development of the orally concentrated polyp nervous system. Therefore, reorganization of the ectodermal nervous system at metamorphosis appears to be an ancestral condition in Cnidaria. In this paper, we have discovered that the early-born RPamide-positive sensory nerve net of the aboral ectoderm is removed at metamorphosis by programmed cell death. We also note that early-born GLWamide- and RFamide-positive sensory cells in the aboral ectoderm similarly disintegrate by undergoing DNA fragmentation at metamorphosis (Figure 5). Hence, we suggest that sea anemones have in fact retained the ancestral pattern of cnidarian neural development to undergo reorganization of the peptidergic ectodermal nervous system at the planula-polyp transition.

## Data Availability Statement

The raw data supporting the conclusions of this article will be made available by the authors, without undue reservation, to any qualified researcher.

## Author Contributions

NN conceived and designed the study. HZ and NN carried out the experiments and drafted the manuscript.

### Conflict of Interest

The authors declare that the research was conducted in the absence of any commercial or financial relationships that could be construed as a potential conflict of interest.

## Supplementary Material

Supplemental Figure 1 Reverse Transcriptase (RT) PCR of RPamide-encoding genes. cDNA PCR results showing the presence of Nv244953 transcripts (expected size: 291 bp) and the absence of Nv37852 transcripts (expected size: 207 bp). “–” denotes a negative control without cDNA. 100 bp ladders were used. cDNA was generated from RNA extracted from planulae and primary polyps. Primer sequences used for PCR are: “244953” Forward 5′-GCTCGGTACAGAGCCGAAACCTGAGACAC-3′, Reverse 5′-CATGGGCAACGGTCAGCGGCAGATCGATG-3′, “37852” Forward 5′-GCCTTGGTCGTGTTGCCTTCGCCCTGGTC-3′, “2” Reverse 5′-CTATACGACCAGGGCGAGGCTACACGACC-3′.

Supplemental Figure 2 *In situ* hybridization using a sense riboprobe. Z-projections of confocal sections of *Nematostella vectensis* at mid-planula **(A,B)** and tentacle-bud/primary polyp **(C,D)** stages, labeled with a sense NvRPa riboprobe (“sense probe”) and an antibody against acetylated ∂-tubulin (“acTub”). All panels are side views of animals with the oral opening facing up. **(A,C)** show superficial planes of section at the level of the ectoderm; **(B,D)** show longitudinal sections through the center. No cell-type-specific staining is observable. Scale bar: 50 μm.

Supplemental Figure 3 CRISPR mutagenesis and validation of an anti-RPamide antibody. **(A)** Schematic view of the NvRPa locus. **(B)** Genomic DNA nested PCR results of Cas9-injected wildtype control embryos (“WT”) and F0 embryos injected with NvRPa locus-specific sgRNAs together with Cas9 (“RPa”). Results of secondary PCR are shown. **(C,D)** Medial-level confocal sections of Cas9-injected wildtype control **(C)** and NvRPa F0 mutant **(D)** mid-planulae at 4 day-post-fertilization (dpf), labeled with an anti-RPamide (“RPa”). Nuclei are labeled with DAPI. Oral side is up. **(E)** A histogram showing the number of anti-RPamide-positive neurons in Cas9-injected wildtype control animals (“WT”; *n* = 5) and NvRPa F0 mutants (“CR-RPa”; *n* = 5) at the 4 dpf mid-planula stage. In **(A)**, a blue bar shows a predicted translation start site; a red bar shows a predicted translation termination site; yellow bars show predicted RPamide-encoding regions as in Figure 1. Purple arrowheads show sgRNA target sites. Blue arrows mark regions targeted in the PCR analysis shown in **(B)**. Note in B that genomic PCR of the WT embryo shows the expected size of PCR fragments (1690 bp for secondary nested PCR), while F0 mutant embryos show additional bands of smaller sizes (arrowhead), indicating that targeted deletions of different sizes have occurred mosaically in each embryo. Over 88% of PCR genotyped embryos showed clear mutant bands (i.e., PCR fragments that are smaller than expected from a wildtype allele; *n* = 34). DNA sequencing of mutant bands (e.g., arrowhead) has confirmed that excision of RPamide-encoding regions can be induced by this CRISPR-mediated mutagenesis approach. In **(C,D)**, arrowheads show anti-RPa-positive neurons. Note in **(D)** that an anti-RPa-positive cell is observed in the endoderm (en) but not in the ectoderm (ec). In **(E)**, ^*^ denotes a statistically significant difference (α = 0.05). Scale bar: 50 μm.

Supplemental Figure 4 Immunostaining with a preadsorbed anti-RPamide antibody. Z-projections of confocal sections of *Nematostella vectensis* at mid-planula **(A,C)** and primary polyp **(B,D)** stages, labeled with an anti-RPamide antibody (“anti-RPa”; **A,B**) and an anti-RPamide antibody preadsorbed with Nv-RPamide IV (CEDSSNYEFPPGFHRPamide) (“Preadsorbed anti-RPa”; **C,D**). All panels are side views of animals with the oral opening facing up. **(A,C)** show longitudinal sections through the center; **(B,D)** show sections through the entire animals. No cell-type-specific staining is observable with the preadsorbed antibody, indicating that the anti-RPamide indeed reacts with Nv-RPamide IV. Scale bar: 50 μm.

Supplemental Figure 5 GLWamide- and RFamide-immunoreactive sensory cells of the aboral ectoderm undergo apoptosis at metamorphosis. Z-projections of confocal sections of *Nematostella vectensis* at the tentacle-bud stage, labeled with antibodies against acetylated ∂-tubulin (“acTub”) and GLWamide [“GLWa”; (5)] or RFamide (“RFa”; unpublished). DNA fragmentation is detected by TUNEL assay (“TUNEL”). All panels show side views of the animal with the oral opening facing up; **(A,C)** represent superficial planes of section at the level of surface ectoderm, while **(B,D)** depict longitudinal sections near the center. Boxed areas are magnified in insets; note that in insets in **(B,D)** the apical surface of the ectoderm faces up. Arrowheads point to TUNEL-positive DNA fragmentation, evidencing programmed cell death. Scale bar: 50 μm.
