# Supplementary figures and images for: Expression Analysis of Cnidarian-Specific Neuropeptides in a Sea Anemone Unveils an Apical-Organ-Associated Nerve Net That Disintegrates at Metamorphosis
Source: Front Endocrinol (Lausanne). 2020 Feb 19;11:63. doi: 10.3389/fendo.2020.00063 (PMC7042181; doi:10.3389/fendo.2020.00063)

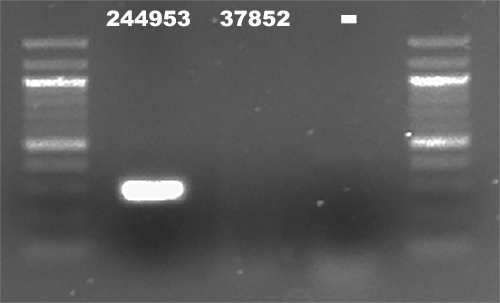

Supplement: Supplemental Figure 1 — Reverse Transcriptase (RT) PCR of RPamide-encoding genes. cDNA PCR results showing the presence of Nv244953 transcripts (expected size: 291 bp) and the absence of Nv37852 transcripts (expected size: 207 bp). “–” denotes a negative control without cDNA. 100 bp ladders were used. cDNA was generated from RNA extracted from planulae and primary polyps. Primer sequences used for PCR are: “244953” Forward 5′-GCTCGGTACAGAGCCGAAACCTGAGACAC-3′, Reverse 5′-CATGGGCAACGGTCAGCGGCAGATCGATG-3′, “37852” Forward 5′-GCCTTGGTCGTGTTGCCTTCGCCCTGGTC-3′, “2” Reverse 5′-CTATACGACCAGGGCGAGGCTACACGACC-3′. [file Image_1.tif]

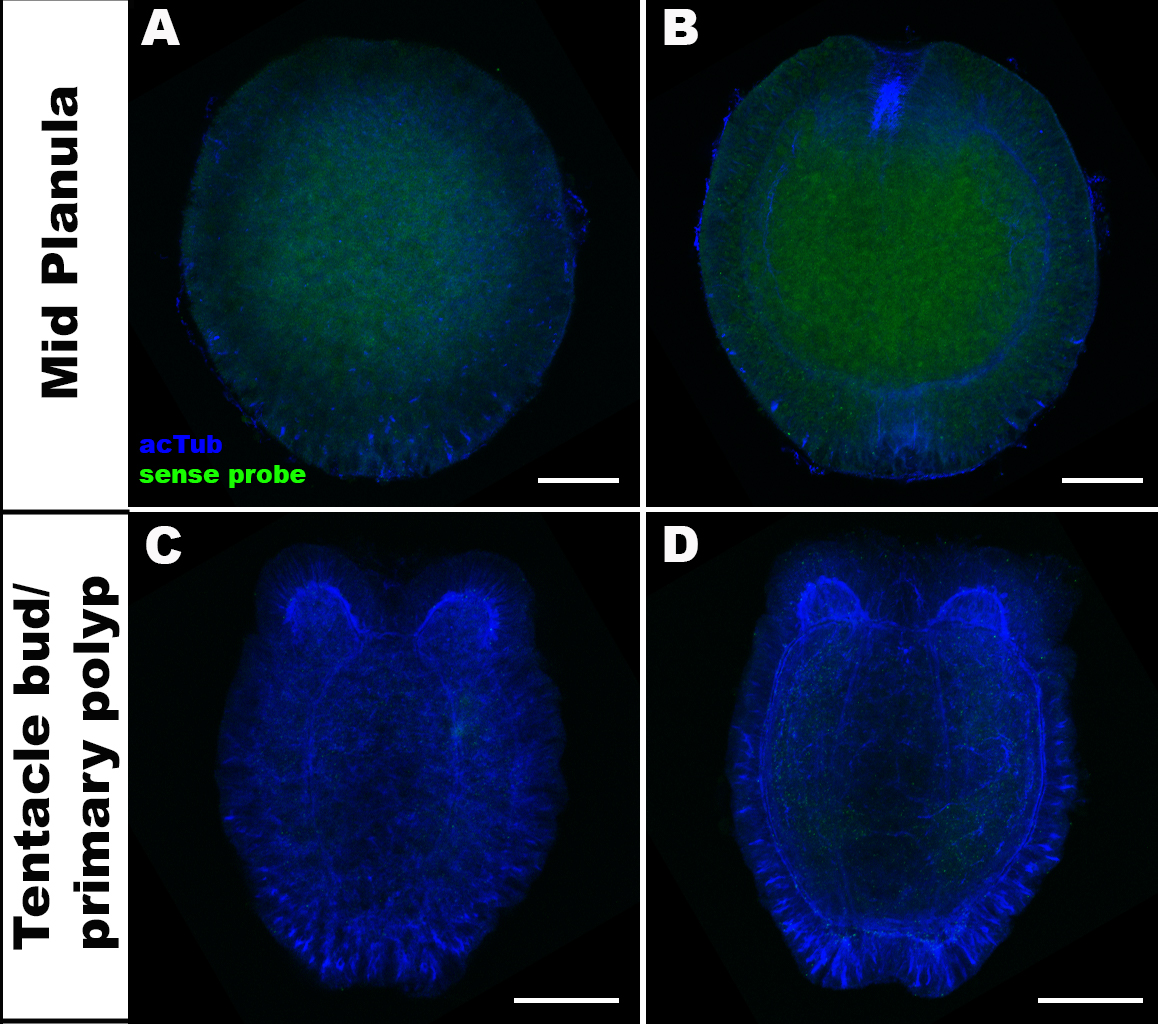

Supplement: Supplemental Figure 2 — In situ hybridization using a sense riboprobe. Z-projections of confocal sections of Nematostella vectensis at mid-planula (A,B) and tentacle-bud/primary polyp (C,D) stages, labeled with a sense NvRPa riboprobe (“sense probe”) and an antibody against acetylated ∂-tubulin (“acTub”). All panels are side views of animals with the oral opening facing up. (A,C) show superficial planes of section at the level of the ectoderm; (B,D) show longitudinal sections through the center. No cell-type-specific staining is observable. Scale bar: 50 μm. [file Image_2.JPEG]

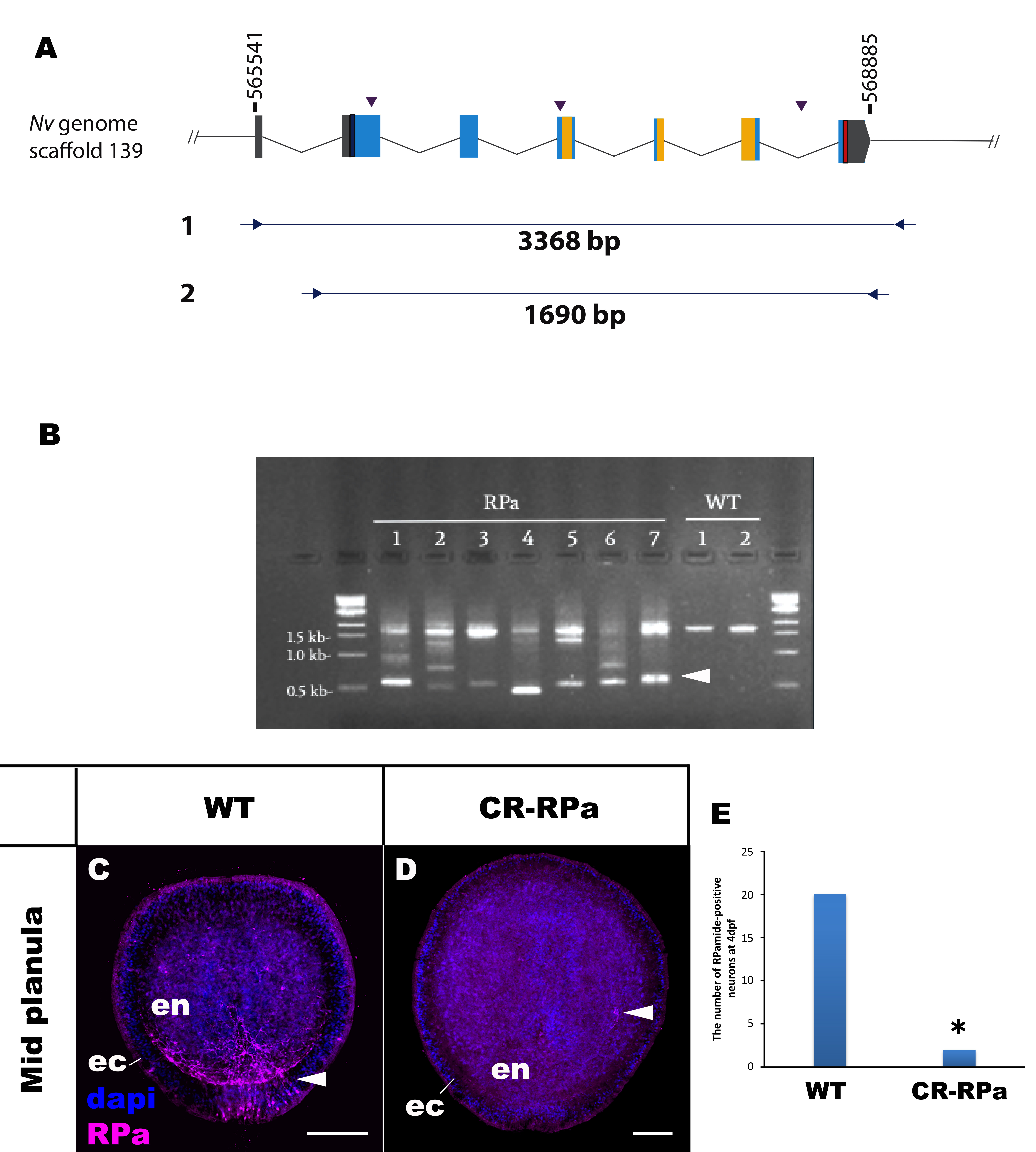

Supplement: Supplemental Figure 3 — CRISPR mutagenesis and validation of an anti-RPamide antibody. (A) Schematic view of the NvRPa locus. (B) Genomic DNA nested PCR results of Cas9-injected wildtype control embryos (“WT”) and F0 embryos injected with NvRPa locus-specific sgRNAs together with Cas9 (“RPa”). Results of secondary PCR are shown. (C,D) Medial-level confocal sections of Cas9-injected wildtype control (C) and NvRPa F0 mutant (D) mid-planulae at 4 day-post-fertilization (dpf), labeled with an anti-RPamide (“RPa”). Nuclei are labeled with DAPI. Oral side is up. (E) A histogram showing the number of anti-RPamide-positive neurons in Cas9-injected wildtype control animals (“WT”; n = 5) and NvRPa F0 mutants (“CR-RPa”; n = 5) at the 4 dpf mid-planula stage. In (A), a blue bar shows a predicted translation start site; a red bar shows a predicted translation termination site; yellow bars show predicted RPamide-encoding regions as in Figure 1. Purple arrowheads show sgRNA target sites. Blue arrows mark regions targeted in the PCR analysis shown in (B). Note in B that genomic PCR of the WT embryo shows the expected size of PCR fragments (1690 bp for secondary nested PCR), while F0 mutant embryos show additional bands of smaller sizes (arrowhead), indicating that targeted deletions of different sizes have occurred mosaically in each embryo. Over 88% of PCR genotyped embryos showed clear mutant bands (i.e., PCR fragments that are smaller than expected from a wildtype allele; n = 34). DNA sequencing of mutant bands (e.g., arrowhead) has confirmed that excision of RPamide-encoding regions can be induced by this CRISPR-mediated mutagenesis approach. In (C,D), arrowheads show anti-RPa-positive neurons. Note in (D) that an anti-RPa-positive cell is observed in the endoderm (en) but not in the ectoderm (ec). In (E), * denotes a statistically significant difference (α = 0.05). Scale bar: 50 μm. [file Image_3.JPEG]

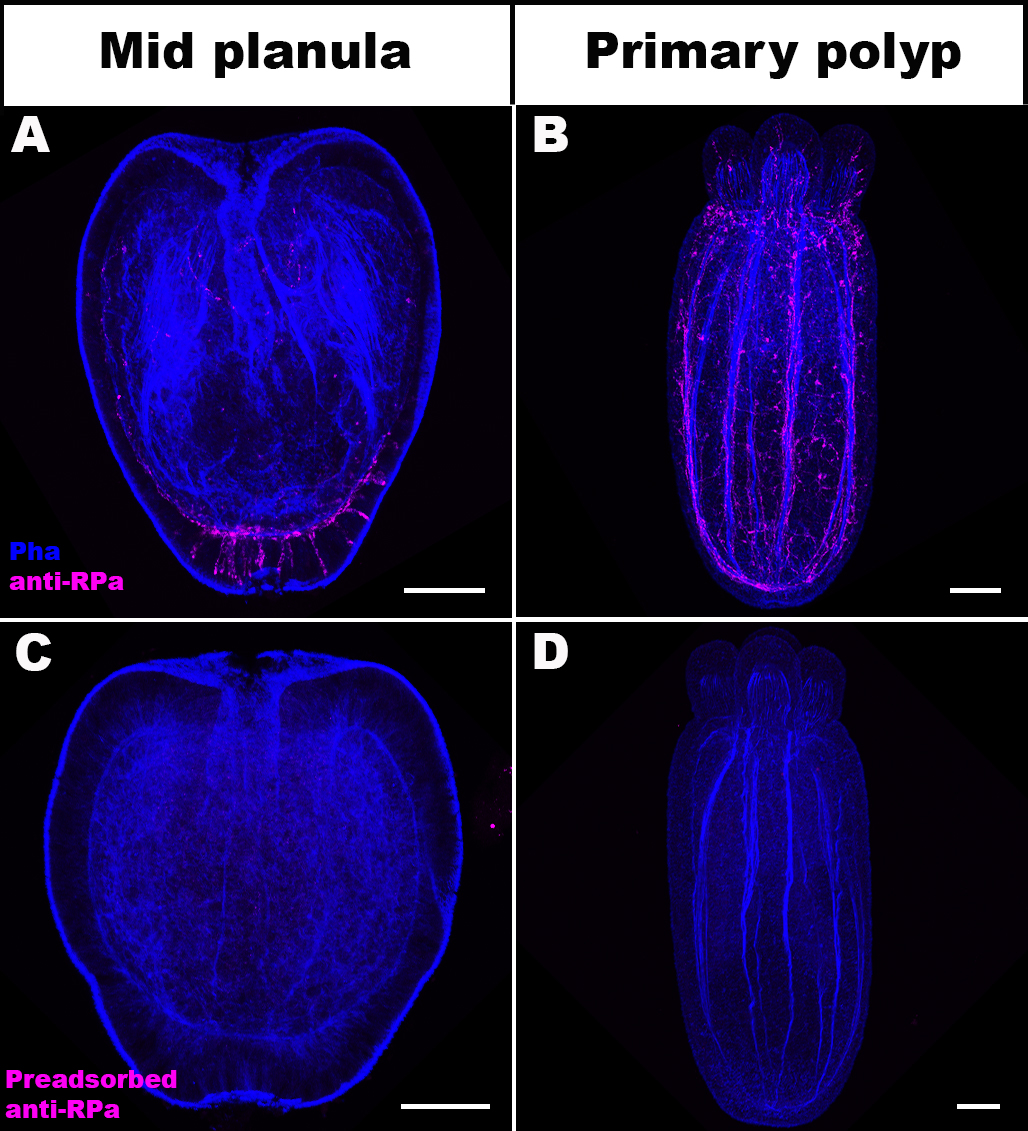

Supplement: Supplemental Figure 4 — Immunostaining with a preadsorbed anti-RPamide antibody. Z-projections of confocal sections of Nematostella vectensis at mid-planula (A,C) and primary polyp (B,D) stages, labeled with an anti-RPamide antibody (“anti-RPa”; A,B) and an anti-RPamide antibody preadsorbed with Nv-RPamide IV (CEDSSNYEFPPGFHRPamide) (“Preadsorbed anti-RPa”; C,D). All panels are side views of animals with the oral opening facing up. (A,C) show longitudinal sections through the center; (B,D) show sections through the entire animals. No cell-type-specific staining is observable with the preadsorbed antibody, indicating that the anti-RPamide indeed reacts with Nv-RPamide IV. Scale bar: 50 μm. [file Image_4.JPEG]

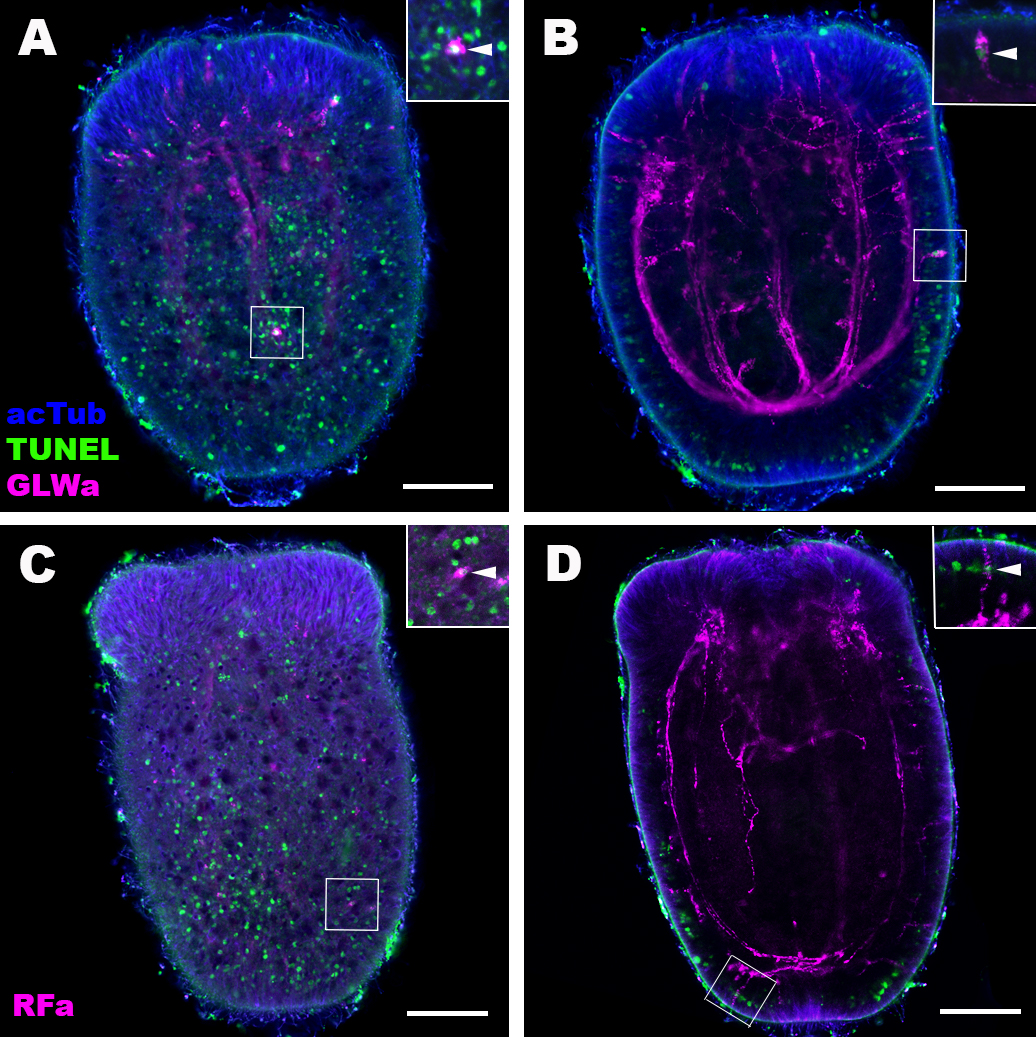

Supplement: Supplemental Figure 5 — GLWamide- and RFamide-immunoreactive sensory cells of the aboral ectoderm undergo apoptosis at metamorphosis. Z-projections of confocal sections of Nematostella vectensis at the tentacle-bud stage, labeled with antibodies against acetylated ∂-tubulin (“acTub”) and GLWamide [“GLWa”; (5)] or RFamide (“RFa”; unpublished). DNA fragmentation is detected by TUNEL assay (“TUNEL”). All panels show side views of the animal with the oral opening facing up; (A,C) represent superficial planes of section at the level of surface ectoderm, while (B,D) depict longitudinal sections near the center. Boxed areas are magnified in insets; note that in insets in (B,D) the apical surface of the ectoderm faces up. Arrowheads point to TUNEL-positive DNA fragmentation, evidencing programmed cell death. Scale bar: 50 μm. [file Image_5.JPEG]
